# NUCLEAR FACTOR Y Transcription Factors Have Both Opposing and Additive Roles in ABA-Mediated Seed Germination

**DOI:** 10.1371/journal.pone.0059481

**Published:** 2013-03-19

**Authors:** Roderick W. Kumimoto, Chamindika L. Siriwardana, Krystal K. Gayler, Jan R. Risinger, Nicholas Siefers, Ben F. Holt

**Affiliations:** Department of Microbiology and Plant Biology, University of Oklahoma, Norman, Oklahoma, United States of America; University of Texas at Austin, United States of America

## Abstract

In the model organism *Arabidopsis thaliana* the heterotrimeric transcription factor NUCLEAR FACTOR Y (NF-Y) has been shown to play multiple roles in facilitating plant growth and development**.** Although NF-Y itself represents a multi-protein transcriptional complex, recent studies have shown important interactions with other transcription factors, especially those in the bZIP family. Here we add to the growing evidence that NF-Y and bZIP form common complexes to affect many processes. We carried out transcriptional profiling on *nf-yc* mutants and through subsequent analyses found an enrichment of bZIP binding sites in the promoter elements of misregulated genes. Using NF-Y as bait, yeast two hybrid assays yielded interactions with bZIP proteins that are known to control ABA signaling. Accordingly, we find that plants mutant for several NF-Y subunits show characteristic phenotypes associated with the disruption of ABA signaling. While previous reports have shown additive roles for *NF-YC* family members in photoperiodic flowering, we found that they can have opposing roles in ABA signaling. Collectively, these results demonstrated the importance and complexity of NF-Y in the integration of environmental and hormone signals.

## Introduction

Successful acclimation of plants to temporal environmental changes requires the integration of multiple intersecting signals. These environmental signals, such as changing light and water availability, profoundly modify growth and developmental programs. Phytohormones often play a central role in these responses and recent studies identified several integrators of light and hormone signaling pathways (reviewed in [1] and [2]). For example, the basic leucine zipper (bZIP) transcription factor ELONGATED HYPOCOTYL 5 (HY5) has been intensely studied for its roles in light regulated development, but was only recently found to additionally mediate abscisic acid (ABA) signaling in germinating seeds [3]. Thus, pathway integrators can act as hubs for multiple environmental inputs, but how they coordinate these variable inputs to generate unique transcriptional outputs remains unknown [2].

One possibility for coordinating multiple environmental inputs is through interactions with combinatorial transcription factors. Increasing evidence suggests that the combinatorial transcription factor NUCLEAR FACTOR Y (NF-Y) plays important roles in facilitating plant responses to various environmental signals and hormones [4]–[7]. In fact, like HY5, NF-Y complexes can regulate both light signaling (blue light) and hormone perception (ABA, [7]). NF-Y transcription factors bind at *CCAAT cis*-elements and function as trimeric complexes consisting of three distinct protein subunits, NF-YA (NF-Y, subunit A), NF-YB, and NF-YC [8]. These individual subunits are each encoded by small gene families found throughout the plant lineage – e.g., *Arabidopsis thaliana* (Arabidopsis) has 36 *NF-Y* encoding genes (10 *NF-YA*, 10 *NF-YB*, and 10 *NF-YC* [9]–[11]). Because the mature DNA-binding NF-Y complex is thought to contain only one of each subunit type, hundreds of unique combinations are theoretically possible. Thus, unique combinations of NF-Y transcription factors may provide a flexible system for fine-tuning the integration of environmental signals to transcriptional outputs [9], [12], [13].

NF-Y are known to regulate a variety of developmental phenotypes and stress responses. For example, NF-YB and NF-YC subunits regulate photoperiod-dependent flowering [5], [6], [13]–[16] and overexpression of both NF-YA and NF-YB proteins can confer drought tolerance in plants [17], [18]. In the legumes *Phaseolus vulgaris* and *Medicago truncatula*, specific NF-YA and NF-YC subunits are necessary for the development of nitrogen fixing nodules [19]–[21]. Other studies have implicated NF-Ys in embryogenesis, light perception, unfolded protein responses (UPR), and photosynthesis [7], [22]–[27].

Although NF-Y itself represents a multi-protein complex that can independently integrate multiple signals, recent studies have shown additional interactions with other transcription factor families, especially those in the bZIP family. Yamamoto *et al.* [4] demonstrated that central regulators of embryogenesis, LEAFY COTYLEDON 1 (LEC1/NF-YB9) and LEC1-LIKE (L1L/NF-YB6), interact with bZIP67 to regulate the expression of genes with ABA-responsive elements (ABRE) in their promoters. More recently, Liu and Howell [25] proposed a model where a complex of bZIP28, NF-YA4, NF-YB3, and NF-YC2 binds to endoplasmic reticulum stress response elements (ERSE) to regulate genes related to the unfolded protein response (UPR). In both cases, full activation of target promoters required the presence of NF-Y and bZIP components. Therefore, the complexity of NF-Y transcription factors can be further increased through interactions with bZIP proteins.

Like NF-Ys, bZIPs are also found throughout the eukaryotic lineage. In Arabidopsis bZIP proteins represent a large family containing 75 members in ∼20 phylogenetically related sub-groups [28]. Similar to NF-Y, bZIP proteins are involved in diverse processes ranging from environmental stress tolerance to development. Group A bZIP proteins are particularly important for the integration of environmental stress signals (e.g., drought) with hormone-related responses (especially ABA, [29]). Examples of Group A bZIP proteins include the previously discussed bZIP67, as well as ABSCISIC ACID INSENSITIVE 5 (ABI5) and the ABA RESPONSE ELEMENT BINDING PROTEINS/ABA BINDING FACTORS 1–4 (AREB/ABF1–4). Mutations in *ABI5* allow developing seeds to germinate and grow on normally restrictive levels of ABA [30]. Mutant and overexpression lines for *ABF1-4* show characteristic morphological and molecular phenotypes typically associated with ABA responses, including altered growth, transpiration rates, and seed germination in response to abiotic stress [31]. Although not in Group A, the light and ABA-signaling integrator HY5 is also a bZIP protein. HY5 directly regulates the expression of *ABI5* and *hy5* mutants have reduced sensitivity to ABA in a variety of assays, including seed germination and root growth [3].

Previously, we showed that *NF-YC3*, *NF-YC4*, and *NF-YC9* have overlapping roles in photoperiod-dependent flowering [16]. In the current study we used transcription profile analysis of *nf-yc3 nf-yc4 nf-yc9* triple mutants to investigate other pathways potentially affected by these genes. This analysis revealed an over-representation of *cis*-elements with putative bZIP binding sites. We hypothesized that *NF-Y* mutants would share phenotypic similarities to various *bZIP* mutants and tested this idea by assaying seed germination, seedling greening, and root elongation for abnormalities as well as quantitative reverse transcriptase polymerase chain reaction analyses (qPCR) on known bZIP regulated genes. We found that various mutant and overexpression combinations of *NF-YB* and *NF-YC* genes significantly altered characteristic morphological and molecular phenotypes associated with ABA signaling. Additionally, we found that NF-Y subunits from the same family can have opposing roles in a given process. Overall, these data suggested that NF-Y and bZIP transcription factors coordinately regulate ABA-related phenotypes.

## Results

### Misregulated Genes in *nf-yc* Mutants are Enriched for bZIP Binding Sites

We previously used qPCR and microarray analyses to demonstrate that *FLOWERING LOCUS T* (*FT*) was downregulated in the 10 day-old seedlings of long day grown *nf-yc3 nf-yc4 nf-yc9* triple mutants (hereafter *nf-yc triple*) [16]. To generate new hypotheses and extend the *nf-yc triple* analysis here, we further examined this published microarray data by searching for over-represented promoter motifs within the 5′ upstream sequences of the misregulated gene set (both up and down regulated genes, p<0.05 without false discovery rate correction; see Materials and Methods). This set consisted of 83 nuclear-encoded genes that were at least 1.5 fold misregulated in the *nf-yc triple*. Initially, we used the online software package Athena to search this gene set for enriched promoter motifs (http://www.bioinformatics2.wsu.edu/cgi-bin/Athena/cgi/home.pl, [32]).

Using the Athena analysis suite, three statistically over-represented promoter motifs were discovered (Table I). All three were bZIP-binding sites with the core-conserved sequence *ACGT*. The most highly significant motif was the 3.3-fold over-represented *CACGTG* motif, also called the G-box [33]. The G-box is found in the promoters of many light responsive genes and is a known binding site for HY5 [34]–[36]. The other two significantly enriched sequences were related to the ABRE and also have the *ACGTG* portion of the G-box. ABREs are also bound by bZIP proteins, specifically the Group A bZIP transcription factors AREB/ABF1-4 [28], [37], [38]. These data are consistent with a previous report that L1L (NF-YB6) [24], [39] physically interacts with the Group A bZIP protein bZIP67 to regulate ABRE containing promoters [4]. Also of note in Table I is the enrichment of the unfolded protein response (UPR) element, as identified in animal systems [40]. Although just missing statistical significance in our dataset, Liu and Howell recently showed that NF-Ys can physically interact with bZIP28 and affect the expression of genes with the UPR promoter element [25].

**Table 1:** Over-represented *cis* motifs in promoters of genes misregulated in *nf-y triple* mutants.

Motif Name	Sequence	Database	% in set (#)	% in genome (#)	Pvalue
**CACGTG**	CACGTG	PLACE^a^	30% (25)	9%	<10e−7
		(S000042)		(2797)	
**ABRE like**	BACGTGKM	AtcisDB^b^	28%	12%	<10e−4
			(24)	(3681)	
**ACGT ABRE**	ACGTGKC	PLACE	24%	9%	<10e−4
		(S000394)	(20)	(2788)	
**UPR Motif II**	CCNNNNNNNNNNNNCCACG	PLACE	8%	2%	0.001
		(S000426)	(7)	(613)	

a Database of Plant Cis-acting Regulatory DNA Elements, http://www.dna.affrc.go.jp/PLACE/ [80].

b Arabidopsis cis-regulatory element database, http://arabidopsis.med.ohio-state.edu/AtcisDB/ [81], [82].

**Figure 1:**
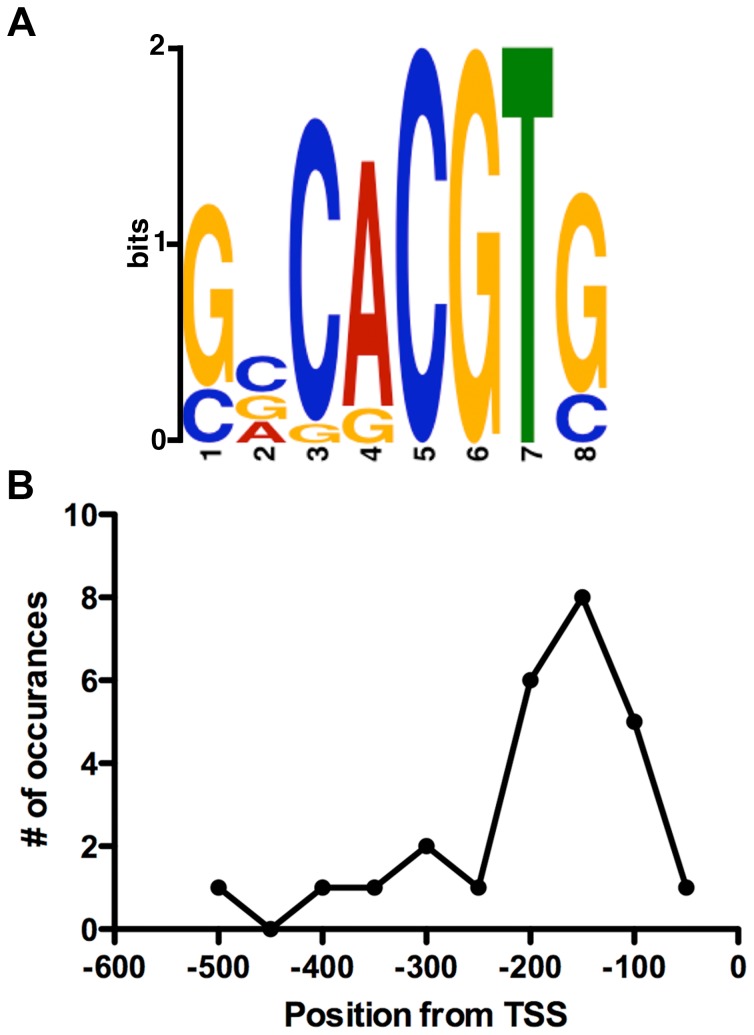
Misregulated genes in the *nf-yc triple* mutant have ABRE-like promoter elements. A) ABRE-like motif discovered through MEME analysis. B) Positional distribution of MEME motif within the promoter set. TSS - transcriptional start site. To help assess the relationships between Arabidopsis NF-Y proteins discussed here and below, note that phylogenetic trees were previously published [9], [76], [77].

In addition to Athena, we used MEME (Multiple Em for Motif Elicitation, http://meme.nbcr.net) to search for shared motifs in the misregulated gene set [41], [42]. MEME uses a Hidden-Markov model and is not constrained by searching for known motifs (i.e., it can find both known and novel motifs). Three low E-value motifs were discovered and each was tested for resemblance to previously identified transcription factor binding sites using the TOMTOM motif comparison tool [43]. While two of the motifs showed no clear homology to known binding sites, one showed significant resemblance to known bZIP binding sites, including those bound by ABF, HY5 and EmBP-1 (Figure 1A). Consistent with our previous results, bZIP binding sites were often located between −100 to −200 bp of the transcription start site within the promoters of the misregulated gene set (Figure 1B). These data, as well as several recent reports [4], [25], suggest that NF-Y may have a generalizable relationship with bZIP proteins in the transcriptional regulation of numerous genes.

### Predicted NF-YC Interaction Network Include bZIP Proteins

**Figure 2:**
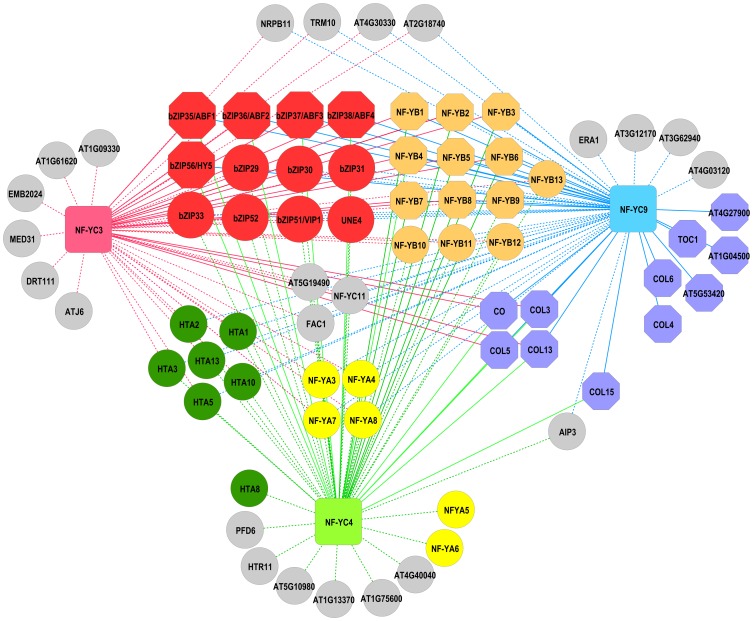
NF-YC3, NF-YC4 and NF-YC9 protein-protein interaction network. Both demonstrated and GeneMANIA predicted protein-protein interaction data for NF-YC3, NF-YC4, and NF-YC9 were visualized using Cytoscape [46]. Predicted physical interactions are depicted as dashed lines, while demonstrated interactions ([6], [16] and this work) are depicted as solid lines. Input nodes NF-YC3, NF-YC4 and NF-YC9 are shown as squares. Circle nodes are those predicted data from GeneMANIA [44], [45]. Octagonal nodes represent demonstrated physical interactions (e.g., Y2H, some shown in this report - see below). Related protein nodes are color coded as follows: red-bZIP; blue – CCT; green - H2A; orange/tan - NF-YB; yellow – NF-YA; gray – unclassified interacting proteins. A Microsoft Excel file is available to recreate/manipulate this data (File S1). Common names were used where available - File S1 contains all AGI numbers and references for sources of data.

Bioinformatic analysis of promoter elements enriched on the *nf-yc triple* microarray revealed a possible interaction with bZIP domain containing proteins. To further investigate the predicted protein-protein interaction network of NF-YC3, NF-YC4, and NF-YC9, we used the web-based software GeneMANIA that is designed to identify functional associations [44], [45]. For visualization, individual protein-protein interaction networks from GeneMANIA were integrated into a single network map using the open source software Cytoscape (Figure 2, [46]). In addition to the predicted interaction network provided by GeneMANIA, we included previously published NF-Y by CCT (Constans, Constans-Like, TOC1) protein interactions. We also included bZIP and CCT protein interactions discovered in a yeast two hybrid (Y2H) screen using NF-YC9 as bait and the directed Y2H data shown below ([16] and RWK and BFH unpublished). As expected, all 13 Arabidopsis NF-YB proteins, as well as four of 10 NF-YA proteins, were predicted to interact with the NF-YC proteins. In addition to the previously described interactions with NF-YA, NF-YB and CCT proteins [5], [6], [16], there were two other large classes of proteins represented on the network map - histone 2A and bZIP containing proteins. These data further support the bZIP binding site enrichment found in the *nf-yc triple* microarray gene set, as well as previously reported interactions between NF-Y and bZIP proteins.

### NF-Y can Physically Interact with ABA Related bZIP Proteins

Relevant to the promoter analyses, NF-YC interaction network, and hypothesis that bZIP/NF-Y interactions are common, we isolated bZIP proteins in Y2H screens using both NF-YB2 and NF-YC9 as bait. When NF-YB2 was used as the bait, we isolated and sequenced 42 positive clones. Five of the positive clones were either bZIP1 (2 independent clones) or ABF3 (3 independent clones). Further, a screen using NF-YC9 as bait yielded a single clone of ABF2 out of 200 sequenced positives. Although a single clone of ABF2 appears low, it should be noted that 78% of the sequenced positive clones from the NF-YC9 screen were either NF-YB or CCT proteins (155 out of 200).

To further validate and extend the bZIP/NF-Y interactions from the Y2H library screens, we independently cloned full-length versions of five bZIP genes with known roles in ABA responses: ABF1, ABF2, ABF3, ABF4 and HY5. Each of these was tested for interaction with a panel of NF-YB and NF-YC proteins in directed Y2H assays. For the NF-YC proteins, we chose the members of the *nf-yc triple* used in the above microarray analysis (NF-YC3, NF-YC4, and NF-YC9). These three proteins are known to genetically interact in an overlapping manner to control *FT* expression and presumably they are interchangeable in the NF-Y complexes controlling *FT* transcription [16]. Likewise, we tested NF-YB2 and NF-YB3 against the panel of bZIPs. NF-YB2 and NF-YB3 are known *in vivo* interactors with NF-YC3 and NF-YC4 and also have overlapping functions in the control of *FT* expression [14], [16]. Finally, NF-YB1 was tested because of its known roles in drought resistance [18] - i.e., we hypothesized a possible connection to the known roles of the ABF/AREBs in various ABA-related responses, including drought resistance.

**Figure 3:**
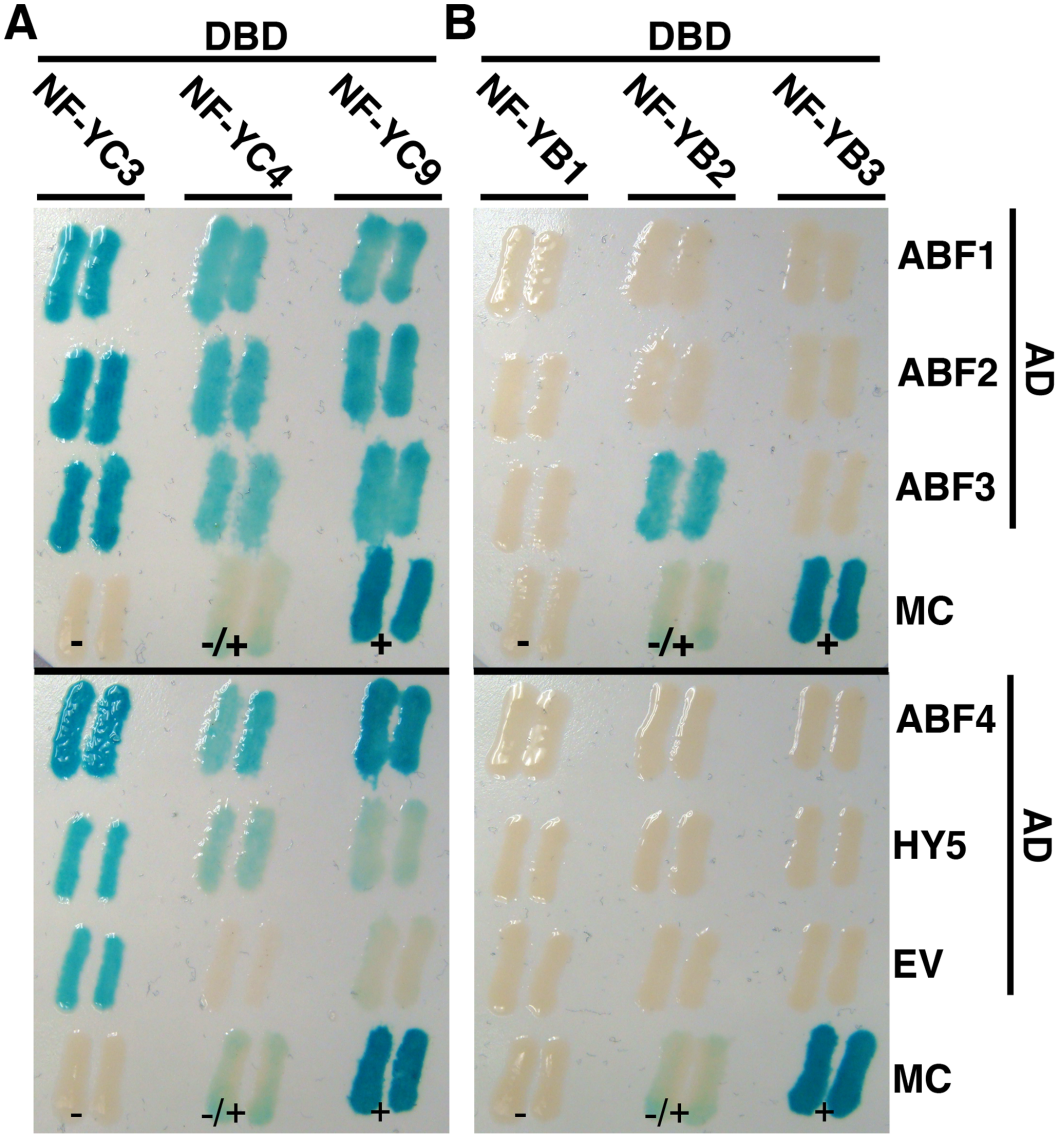
NF-YB and NF-YC subunits interact with bZIP transcription factors. Directed Y2H interactions between NF-YB or NF-YC subunits fused to DNA binding domains (DBD) and select bZIP proteins fused to activation domains (AD). Two independent colonies are shown for the activation of a β-galactosidase reporter gene (similar activation seen for two other reporters). A) NF-YC3, NF-YC4 and NF-YC9 interactions with ABF1-4 and HY5. B) NF-YB1, NF-YB2 and NF-YB3 interactions with ABF1-4 and HY5. MC = manufacturer’s controls (+ = strong positive, +/− = intermediate positive, − = negative), EV = empty vector.

NF-YC4 and NF-YC9 interacted with all five of the tested bZIP proteins (Figure 3A). NF-YC3 also likely interacted with the four ABFs, but this result was difficult to accurately interpret due to previously reported autoactivation ([16], compare to empty vector interaction in Figure 3A). Previous reports indicate that NF-YC subunits also mediate bZIP interactions in fungal and mammalian systems [47], [48], suggesting broad evolutionary conservation of these interactions in the eukaryotic lineage.

Although the NF-YB2 by ABF3 interaction was reproducible in the directed Y2H tests (Figure 3B), no other NF-YB by bZIP interaction was detected. This result was unanticipated because NF-YB2 and NF-YB3 share nearly 70% amino acid identity over their entire proteins. Further, their central conserved domains, the 95AA histone fold motif (HFM), are 93% identical and 100% similar (Figure S1). This led to the hypothesis that regions outside of the conserved domain in NF-YB2 are responsible for its unique ability to interact with ABF3.

**Figure 4:**
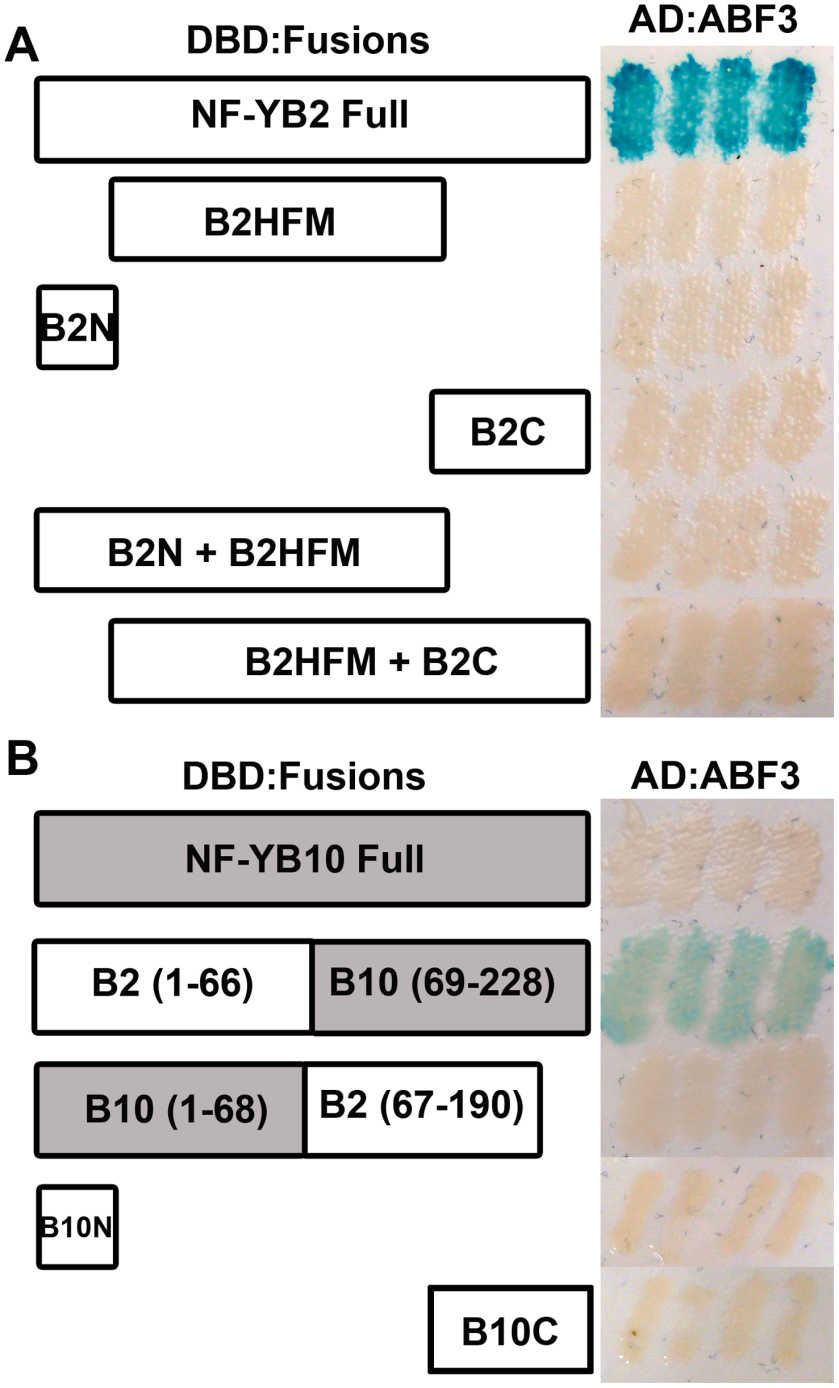
Full length NF-YB2 is required for the ABF3 interaction. Y2H assays were performed between AD:ABF3 and DBD fused to: A) Full length NF-YB2 (AA 1–190), B2HFM (AA 26–121), B2N (AA 1–25), B2C (AA 122–190), B2N+B2HFM (AA 1–122), and B2HFM+B2C (AA 122–190); B) Chimeric constructs - full length NF-YB10 (AA1–228), NF-YB2/NF-YB10, NF-YB10/NF-YB2, NF-YB10N (AA1–27) and NF-YB10C (AA 123–228).

To test this hypothesis we dissected the NF-YB2 protein into three different regions: the N-terminal region up to the conserved domain (Figure 4A, B2N, AA 1–25), the conserved HFM (B2HFM, AA 26–121) and the C-terminal region (B2C, AA 122–190). Constructs containing each of the regions were tested in Y2H for interaction with ABF3. None of the partial NF-YB2 clones showed interaction with ABF3 (Figure 4A), although the conserved histone fold motif of NF-YB2 still retained the ability to interact with NF-YC proteins (data not shown). This led to the hypothesis that one of the ends of NF-YB2, in conjunction with the HFM, could drive the interaction with ABF3. Clones were generated from the start of NF-YB2 through the HFM (B2N+B2HFM, AA 1–121) and starting from the first AA of the HFM through the end of the protein (B2HFM+B2C, AA 26–190). Like the partial constructs above, neither of these constructs interacted with ABF3 (Figure 4A). Thus, if the N or C terminal ends were required for NF-YB2 specificity for ABF3 interactions, these appeared to still require the context of the full-length protein.

To explore this latter idea, we created chimeric proteins between NF-YB2 and NF-YB10. We chose NF-YB10 because it has highly divergent terminal ends outside of the HFM and also showed no interaction with ABF3 (Figures 4B, S1). The constructs contained either the N-terminal region of NF-YB2 or NF-YB10 through the first half of the HFM fused to the C-terminal region of the other NF-YB (Figure 4B). Interestingly the NF-YB2 N-terminal/NF-YB10 C-terminal chimeric protein was able to interact with ABF3, although more weakly than the native full length NF-YB2. This is in contrast to the complementary construct (NF-YB10 N-terminal/NF-YB2 C-terminal), which did not interact (Figure 4B). Further, neither constructs containing the NF-YB10 N-terminal (B10N, AA 1–22) or C-terminal (B10C, AA 123–228) regions alone interacted with ABF3. Collectively, these data suggest that the N-terminal domain of NF-YB2 is necessary but not sufficient for the specific interaction with ABF3. A previous report in Arabidopsis also suggested that bZIP28 interactions with NF-Ys were subunit specific (NF-YB3) and additionally required the initial formation of an NF-YB3/NF-YC2 dimer [25]. Thus, additional interactions may be masked in our two way directed tests.

### *NF-YC* have Opposing Roles in ABA Responses

**Figure 5:**
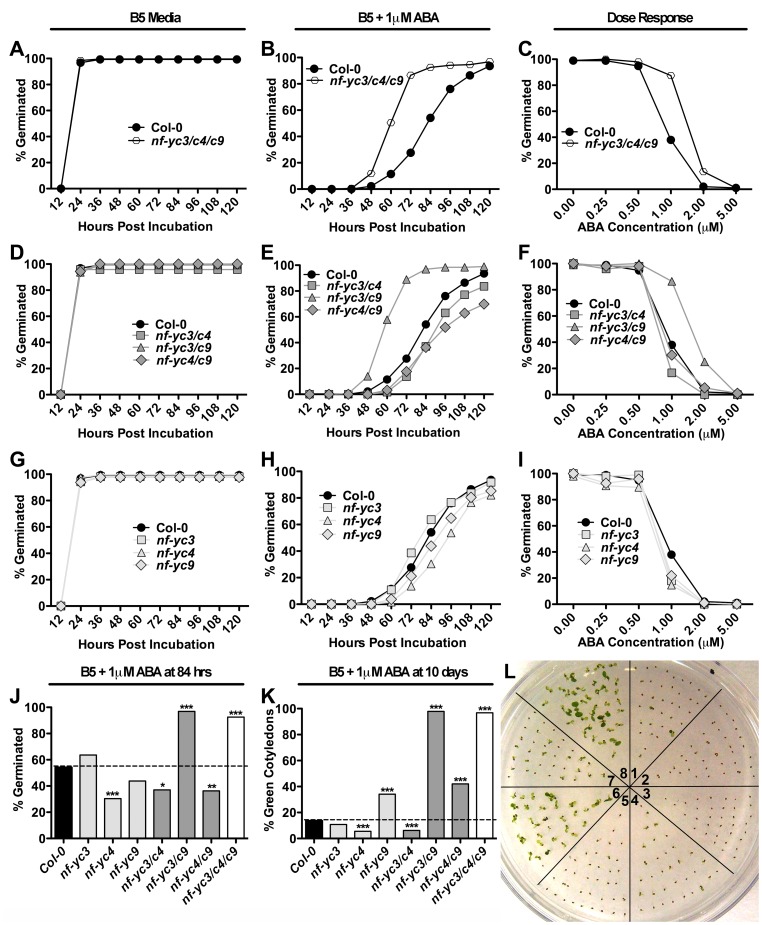
*NF-YC* mutants show opposing phenotypes in response to ABA. A–C) Germination of *nf-yc triple* mutants on B5 media, B5 media with 1 µM ABA, and in response to variable ABA dosage at 84 hours, respectively. D–F) As in A–C for all *nf-yc* double mutant combinations and G–I) single mutants. J) Percent germination for all *nf-yc* mutant combinations on 1 µM ABA at 84 hours. K) Percentage of plants with open green cotyledons at 10 days for all combinations of *nf-yc* mutants. L) Picture of 10 day old seedlings grown on 1 µM ABA; 1 = Col-0, 2 = *nf-yc3*, 3 = *nf-yc4*, 4 = *nf-yc9*, 5 = *nf-yc3/c4,* 6 = *nf-yc3/c9,* 7 = *nf-yc4/c9,* 8 = *nf-yc triple*. Asterisks for J–K represent Fisher’s Exact Test p-values; *p<0.01, **p<0.001, ***p<0.0001. Germination data is a compilation of two experiments (n = 6 replicates per genotype) using independent sets of matched seeds. Each replicate contained at least 30 seeds.

Our bioinformatic analyses and Y2H screens suggest that NF-Y/bZIP interactions may be commonplace. These findings led us to hypothesize that, like many bZIPs, NF-Y are also involved in ABA responses. Warpeha et al. [7] previously demonstrated that several Arabidopsis *nf-y* mutants have delayed germination in the presence of ABA. We extended these analyses by first testing the germination of *nf-y triple* mutants on non-ABA containing media, where no significant differences to control were observed (Figure 5A). Conversely, on media supplemented with ABA, germination inhibition was significantly reduced in *nf-yc triple* mutants compared to Col-0 control plants (Figure 5B–C,J). This result was similar to previous observations of both *abf1* or *hy5* mutants [49], [50].

Although *NF-YC3*, *NF-YC4* and *NF-YC9* have overlapping functionality in photoperiod-dependent flowering, it was unclear whether the same would be true for these ABA related phenotypes. In fact, it was previously shown that *nf-yc4* single mutants were hypersensitive to ABA [7] - the opposite of what we observed for the *nf-yc triple* mutant which includes the same *nf-yc4-1* loss of function allele. Therefore, we tested the germination of all possible single and double mutant combinations of *nf-yc3*, *nf-yc4*, and *nf-yc9* (see Materials and Methods for allele designations).

The response of *nf-yc3 nf-yc9* double mutants on ABA media was non-distinguishable from the *nf-yc triple*, indicating that *NF-YC4* does not normally play a role in repressing ABA-mediated germination (Figure 5D–F,J). As was previously reported [7], *nf-yc4* single mutants were hypersensitive to ABA in our germination assays (Figure 4H,J). Like *nf-yc4*, the *nf-yc3 nf-yc4* and *nf-yc4 nf-yc9* double mutants were also slightly hypersensitive to ABA and germinated later than controls (Figure 5E,J). The opposite is true in *nf-yc triple* mutants where the early germination phenotype of the *nf-yc3 nf-yc9* double mutant is observed (Figure 5B). To extend these ABA analyses beyond germination, we additionally measured cotyledon greening and root elongation.

For cotyledon greening assays, seeds were sown on media containing 1 µM ABA and the percentage of plants with open green cotyledons was scored after 10 days of growth in continuous light [51]. As predicted from the germination data, *nf-yc3 nf-yc9* double and *nf-yc triple* mutants performed much better than controls - after 10 days nearly 100% of these plants had open green cotyledons compared to less than 20% for parental Col-0 (Figure 5K–L). Poor greening for *nf-yc4* single and *nf-yc3 nf-yc4* double mutants also correlated with the germination phenotypes. However, *nf-yc9* single mutants, which showed no significant differences in germination, had a significantly greater number of seedlings that greened (Figure 5K). Additionally the *nf-yc4 nf-yc9* double mutants greened similarly to *nf-yc9* single mutants. This is in contrast to the ABA germination phenotype exhibited by the *nf-yc4 nf-yc9* double mutant where the *nf-yc4* phenotype was observed. Unlike cotyledon greening, *NF-YC* mutants showed no clear root elongation differences on media supplemented with ABA (Figure S2). These observations indicate that *NF-YCs* not only have opposing roles in ABA responses, but also have separable roles in germination and post-germination growth in the above ground plant.

### *NF-YB2* and *NF-YB3* Overexpression Significantly Delays Germination

**Figure 6:**
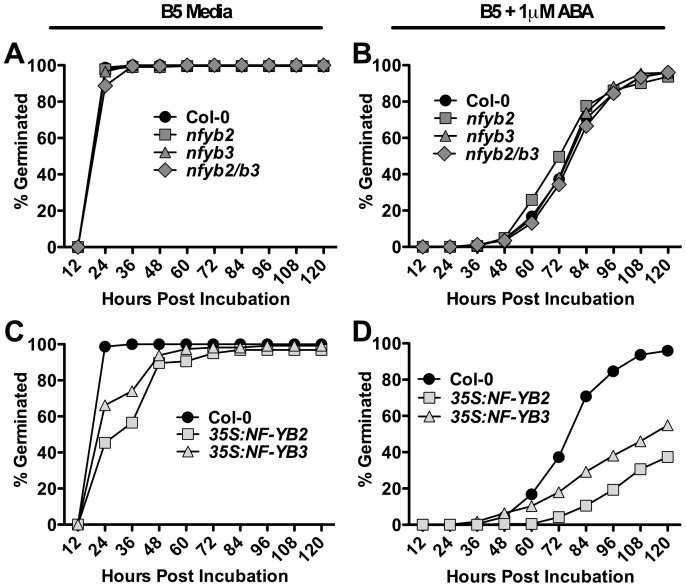
*NF-YB* overexpression results in late germination. A–B) *nf-yb2*, *nf-yb3* and *nf-yb2/b3* double mutants on B5 and B5+1 µM ABA media. C–D) *p35S::NF-YB2* and *p35S::NF-YB3* on B5 and B5+1 uM ABA media. Germination data is compilation of two experiments (total of n = 6 replicates per genotype) using independent sets of matched seeds. Each replicate contained at least 30 seeds. A Fisher’s Exact Test was performed for both mutants (no difference) and overexpression lines at 84 hrs post-incubation. Both overexpression lines were significantly different (p<0.01) from parental Col-0. Separate, independent *NF-YB* overexpression lines had similar results.

Because of the known physical interactions between NF-YB2/NF-YB3 and NF-YC3/NF-YC4/NF-YC9 [16], as well as our demonstration that NF-YB2 can physically interact with ABF3, we additionally tested these NF-YBs in ABA germination assays and root elongation assays. As with *nf-yc3*, *nf-yc4*, and *nf-yc9* mutants, we measured no root growth defects in response to ABA for the *nf-yb2* and *nf-yb3* mutants (Figure S2). Further, we found that neither *nf-yb2* or *nf-yb3* single knockdown mutants, nor *nf-yb2 nf-yb3* double mutants showed any significant alteration in germination on either regular media or ABA-supplemented media (Figure 6A–B; note that the same *nf-yb* alleles have clear late flowering phenotypes [12], [14], [15].

We additionally used the 35S promoter from the cauliflower mosaic virus [52] to overexpress both *NF-YB* genes in transgenic plants [Note that the same experiment with the *NF-YC* genes was not possible due to the consistent inability to obtain stable overexpressing transgenic lines]. Stable, single insertion, third generation *NF-YB* overexpressing plant lines were chosen. When assayed on non-ABA growth media, *p35S::NF-YB2* and *p35S::NF-YB3* seeds reached 100% germination approximately 24 hours later than control plants (Figure 6C). In the presence of 1 µM ABA, *p35S::NF-YB2* and *p35S::NF-YB3* seeds were much later germinating and never reached 100% germination (typically peaking at ∼50% total germination by 120 HAI, Figure 6D). These data demonstrate that *NF-YB2* and *NF-YB3* overexpression can significantly alter seed germination responses, but knockdown data suggests this may not be their normal biological role. These results are similar to what is observed with *abf* mutants, where single loss of function alleles did not show strong phenotypes, but *ABF* overexpression could inhibit germination on ABA media [51], [53], [54]. Thus, higher order mutants combining *nf-yb2*, *nf-yb3*, and additional *nf-yb* knockdown alleles might uncover a biological role in seed germination for *NF-YB2* and *NF-YB3*.

**Figure 7:**
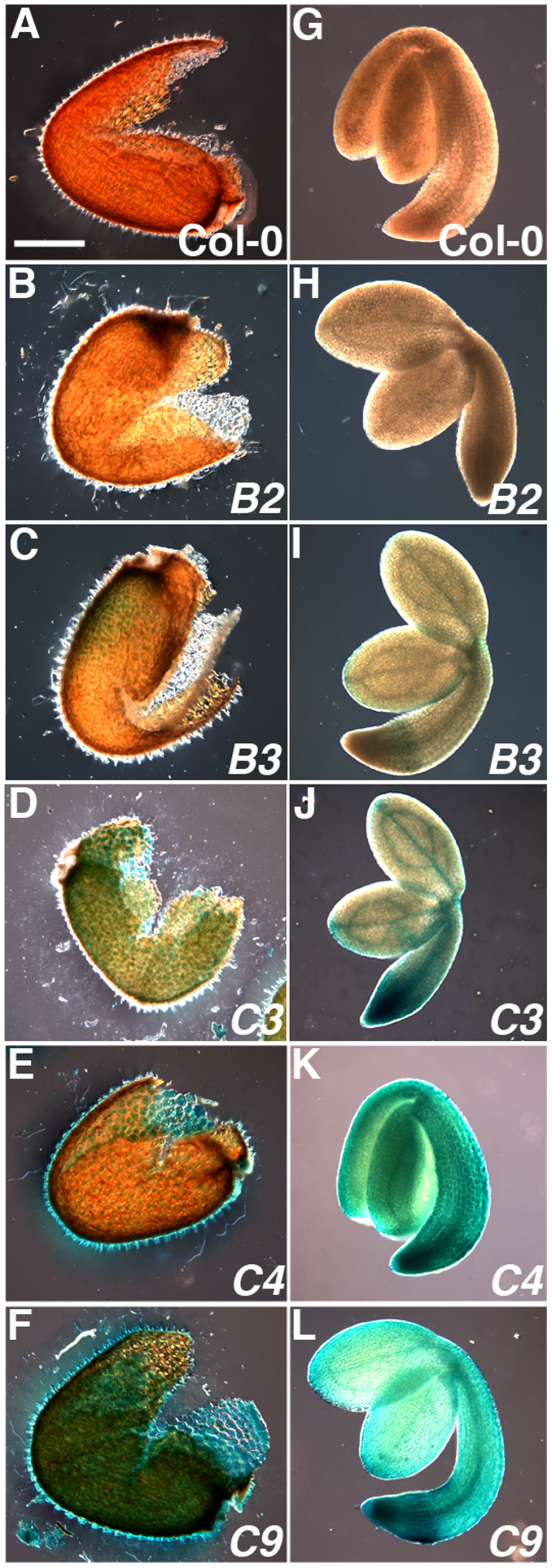
*NF-YC* are strongly expressed in embryos and the endosperm 24 hours post incubation in light. A,G) Col-0, B,H) NF-YB2, C,I) NF-YB3, D,J) NF-YC3 E,K) NF-YC4 F,L) NF-YC9. Scale bar in (A) equals 200 µm.

To help sort through these possibilities, we examined the tissue-specific expression of *NF-YB2* and *NF-YB3* in imbibed seeds using previously described transgenic plant lines expressing the reporter gene β-glucuronidase (GUS) driven by native *NF-Y* promoters (*pNF-Y::GUS* lines, [9]). If these *NF-Y* were likely to be normally involved in seed germination, expression would be expected in either the developing embryos or endosperm. As controls, we also examined the *pNF-Y::GUS* lines for *NF-YC3*, *NF-YC4*, and *NF-YC9* where our loss of function data already clearly demonstrated a biological role in germination. As expected from the *nf-yc* mutant data above, all three *NF-YC* genes were consistently expressed in seeds, including both the embryos and endosperm layer (Figure 7C–F, I–L). Although there were variations in specific expression patterns, all three *NF-YC* had expression throughout the embryonic root with the strongest GUS staining in the meristematic regions. Additionally, staining was typically stronger in the vascular regions of the cotyledons than the remainder of the seed leaf (this contrast was strongest for *NF-YC3*, Figure 7J). However, most *pNF-YB2::GUS* seeds showed no staining in the embryo or endosperm (Figure 7B, H) and *pNF-YB3::GUS* seeds showed only weak and inconsistent staining in the embryo and endosperm (Figure 7C, I). The observed expression patterns for the promoter fusion constructs are consistent with publically available seed microarray data (Figure S3). The addition of ABA did not significantly change the expression patterns for any of the constructs or mRNA patterns from public microarray studies (data not shown). These data support the hypothesis that NF-YB2 and NF-YB3 are not normally the major NF-YB components of the germination-influencing NF-Y complexes. Nevertheless, both can clearly and reproducibly alter germination when ectopically expressed in seeds.

### ABA and Germination Marker Genes have Altered Expression in *NF-Y* Mutants

**Figure 8:**
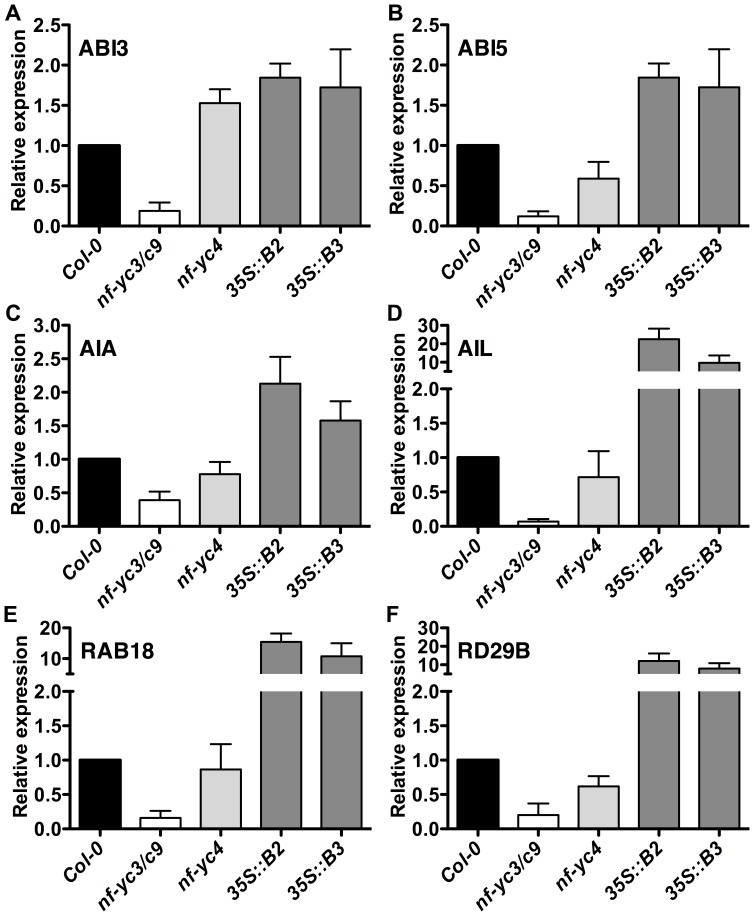
ABA related genes are misregulated in *NF-Y* mutant lines. Gene expression in 24 hr post light incubation seeds analyzed by quantitative RT-PCR for A) *ABI3*, B) *ABI5*, C) *AIA*, D) *AIL*, E) *RAB18*, and F) *RD29B*. For each gene, the expression level in Col-0 was defined as 1. Data represent means and standard deviation of three replicates.

To further test *NF-Y* roles in ABA signaling and germination, we used qPCR to examine transcript levels of several genes that are well-known to be responsive to bZIPs - *ABI3*, *ABI5*, *AIA*, *AIL*, *RAB18*, and *RD29B* [3], [31], [53]. For these assays we examined transcripts from imbibed seeds after 24 h of light exposure (see Materials and Methods). Based on our phenotypic data, we chose to compare the *nf-yc3 nf-yc9* double mutants to the *nf-yc4* single mutant. Additionally, we examined gene expression in the ABA-hypersensitive *p35S::NF-YB2* and *p35S::NF-YB3* lines. Consistent with ABA-insensitivity phenotypes, the germination inhibitors *ABI3* and *ABI5* were downregulated ∼5–10 fold in *nf-yc3 nf-yc9* mutants (Figure 8A–B). The ABA-hypersensitive lines *nf-yc4*, *p35S::NF-YB2*, and *p35S::NF-YB3* all had less dramatic, but increased levels of *ABI3* expression. *ABI5* expression only increased in the *NF-YB* overexpressing lines. Collectively, these data are also consistent with previous research showing that *ABI3* expression is positively regulated by *LEC1* (*NF-YB9*, [55], [56]). For the ABA-regulated genes *AIA*, *AIL*, *RAB18*, and *RD29B*, we consistently measured downregulated expression in the *nf-yc3 nf-yc9* mutants, ranging from −2.5 fold (*AIA*) to −14.3 fold (*AIL*, Figure 8C–F). Once again, the two *p35S::NF-YB* lines consistently had the opposite response. For several genes the differences were quite dramatic. For example, in the *p35S::NF-YB2* transgenic seeds, *AIL* was upregulated 22.5 fold (Figure 8D) and *RAB18* was up 15.5 fold (Figure 8E). None of these ABA-regulated genes were strongly misregulated in *nf-yc4* mutants, although the general trend was unexpectedly slightly down for each gene other than *ABI3*. The lack of strong differences in *nf-yc4* may be due to the relatively weak ABA sensitivity phenotypes reported here and elsewhere [7]. Future experiments examining higher order mutants, especially a double mutant between *NF-YC4* and its apparent paralog *NF-YC1* [9], may improve the resolution of this analysis.

## Discussion

Through bioinformatics and mutant analyses we add to the growing body of evidence that NF-Y and bZIP transcription factors cooperatively regulate similar subsets of genes and, thereby, some of the same plant processes. It also appears likely that NF-Y and bZIP form higher order regulatory complexes capable of integrating inputs from many signaling pathways. Examination of the *cis*-regulatory regions of mis-expressed genes in *nf-yc triple* mutants uncovered an over-representation of G-box and ABRE-like bZIP binding motifs. ABRE and G-box elements are common in the promoters of genes responsive to abiotic stress and light [57], [58]. Studies of ABRE containing promoters demonstrated that single ABRE sequences are not sufficient to induce transcriptional activation and a coupling element is required for induction of ABA-responsive genes [59]. Our data suggests that the NF-Y-bound *CCAAT* box might play the role of the ABRE coupling element for some bZIP-responsive promoters.

In Arabidopsis, Liu and Howell show that a G-box containing promoter (ERSE-I) required intact *CCAAT* and *CACGTG* (G-box) elements for full activation in response to ER stress [25]. Further support that the *CCAAT* box may act as a coupling element to the G-box comes from outside the plant kingdom. Motifs similar to the ABRE were over-represented in *NF-Y* bound mammalian promoters [60]. Further, in response to ER stress, mammalian NF-Y must bind to the *CCAAT* box in ERSE containing promoters before the bZIP protein ATF6 can bind an adjacent G-box-like element [48].

In contrast, Yamamoto *et al.* demonstrated that NF-YB, NF-YC, and bZIP proteins collectively activate the ABRE-containing promoter of *CRUCIFERIN C* (*CRC*), but claim that this activity does not require the *CCAAT* box sequence [4]. Further, the addition of NF-YA subunits to their protoplast assays inhibits *CRC* expression. These data suggest a non-canonical use of the NF-YB/NF-YC dimer where NF-YA subunits are not involved in the final transcriptional complex. In rice, OsMADS18 was shown to interact with an NF-YB/NF-YC dimer, without the NF-YA, suggesting some NF-Y may have evolved to form atypical complexes in plants [27]. Atypical NF-Y complexes (i.e., lacking NF-YA) would be unlikely to bind *CCAAT* boxes because the NF-YA subunit is thought to make all the direct physical contacts with the *CCAAT* nucleotide sequence [61], [62]. Nevertheless, while it is possible that some NF-Y might not bind the actual *CCAAT* box, they were still necessary for bZIP67 activation of CRC expression.

NF-Y and bZIP proteins not only bind the same promoters, but also can physically interact [4], [25]. Here we show that NF-YC subunits interact with different bZIP proteins fairly indiscriminately in directed Y2H assays. This result is consistent with data from animals and fungi where the NF-YCs appear to make the primary contacts with bZIP proteins [47], [48]. While NF-YC proteins indiscriminately bound bZIP proteins in our Y2H assays, the NF-YB proteins were more selective. This suggests that NF-YBs may play a role in discriminating which bZIP binds to an NF-YB/C dimer or the full complex. In addition we demonstrate that regions outside of the highly conserved HFM are likely driving the preference of NF-YB for bZIP partners. This observation is of interest because it is consistent with protoplast assays where only the closely related NF-YB6 and NF-YB9 could interact with bZIP67 to activate the CRC promoter, as well as the finding that bZIP28 only interacts in complexes containing NF-YB3 [4], [25]. Together, these data raise an intriguing hypothesis for the large expansion and maintenance of NF-YB subunits in plants as compared to animal and fungal systems. It is possible that NF-YB have evolved specific interactions with bZIP proteins whereas in other systems the interactions are more general.

In addition to promoter analyses and Y2H assays, we show that NF-Y mutant plants have morphological phenotypes related to ABA signaling and that several well-known marker genes are misregulated in the various mutant backgrounds. Initial germination studies with *nf-yc triple* mutants showed that they are less sensitive to ABA treatments. This was surprising because the *nf-yc4* single mutant, one of the mutants comprising the *nf-yc triple*, was previously reported as being more sensitive to ABA in germination assays [7]. We then tested all possible double mutant combinations as well as the single mutants of *NF-YC3*, *NF-YC4*, and *NF-YC9*. Out of this analysis an epistatic relationship between *NF-YC3*/*NF-YC9* and *NF-YC4* emerged. *nf-yc3/nf-yc9* double mutants showed identical ABA germination phenotypes to *nf-yc triple* mutant plants. This suggests that NF-YC3 and NF-YC9 are interchangeable as negative regulators of germination, and NF-YC4 does not have overlapping functionality in this process. The fact that NF-YC3 and NF-YC9 work as functional equivalents is not surprising as their amino acid sequences are 100% identical throughout the conserved DNA binding and NF-YA/NF-YB interaction domains [16]. NF-YC4 is more divergent in these domains and in agreement with previous reports [7] we demonstrated that *nf-yc4* single mutants are late germinating on ABA-containing media. Epistasis analysis between the late germinating *nf-yc4* mutants and early germinating *nf-yc3/nf-yc9* mutants revealed that *NF-YC3* and *NF-YC9* collectively suppress the role of *NF-YC4* as a positive regulator of seed germination.

The epistasis of NF-YC3/NF-YC9 to NF-YC4 suggests a simple linear genetic pathway where NF-YC4 is upstream and possibly directly regulates NF-YC3 and NF-YC9. Another possibility is that NF-YC3, NF-YC4 and NF-YC9 are acting competitively at single hubs to regulate the ABA germination response. Similar functional antagonism between closely related family members has been seen with the seed specific bZIPs, ABI5 and EEL (Enhanced EM Level, bZIP12, [63]). Mutant analysis revealed that EEL is a negative regulator of embryogenesis-abundant genes and is dependent on the presence of the positive regulator ABI5. Interestingly, instead of a simple linear pathway where EEL regulates ABI5, it was shown that ABI5 and EEL could interact and possibly compete for the same ABRE promoter elements in embryogenesis-abundant genes. It will be interesting to determine if positive and negative regulatory NF-Y complexes can compete for the same elements. In addition it stands to reason that bZIP proteins could also be involved in these complexes.

In an attempt to determine the composition of biologically active NF-Y complexes related to ABA signaling, we additionally tested *nf-yb2* and *nf-yb3* mutants for their roles in germination. Both of their encoded proteins interact *in vivo* with NF-YC3 and NF-YC4 to regulate flowering, but these assays only utilized seedlings [16]. *nf-yb2* and *nf-yb3* loss of function mutants showed no clear difference to controls in ABA germination assays, but gain of function *p35S::NF-YB2* and *p35S::NF-YB3* lines showed strong repression of germination in the presence of ABA. While these gain of function phenotypes suggest a role for NF-YB2 and NF-YB3, their lack of native expression in the endosperm/embryo and the absence of loss of function phenotypes suggest other NF-YB act natively in the germination complex. It is likely that NF-YCs partner with LEC1 and L1L (NF-YB6 and NF-YB9, respectively) as these NF-YB proteins are well-known, embryo-specific regulators [7]. Similar to *nf-yc3 nf-yc9* double mutants, *lec1* mutant plants are less sensitive to ABA in germination assays [23]. In addition, NFYC3, NF-YC4 and NF-YC9 can all interact with LEC1 and L1L in directed Y2H assays [64], [65] and we consistently isolate LEC1 and L1L in library screens using NF-YC9 as bait (BFH, CLS, RWK unpublished data). Although *nf-yc3 nf-yc9* and *lec1* mutants share reduced ABA sensitivity in germination assays, *nf-yc3 nf-yc9* mutants do not have the *lec1* leafy cotyledon or desiccation intolerant phenotypes. This suggests that there may be additional, as yet unidentified NF-YC(s) involved in embryogenesis.

Although NF-Y can interact with bZIP proteins and can regulate the expression of a similar subset of targets, it remains to be determined how and if these complexes form *in planta.* With the recent discovery of the long sought ABA receptor, it has become clear that Group A bZIP transcription factors are directly activated through phosphorylation in the presence of ABA [29], [66]. In contrast to these bZIP proteins, relatively little is known about NF-Y complexes. How NF-Y activity is regulated and what *cis*-regulatory elements they are capable of binding remains to be determined for the plant lineage.

In this study we begin to uncover the complex and sometimes antagonistic roles that NF-Y play in ABA signaling. In addition, we add to the mounting evidence that plant, animal, and fungal NF-Y interact with bZIPs to form multi-protein, transcription-regulating hubs to affect gene expression. Future studies describing the regulation and formation of these complexes will further our understanding of how plants integrate multiple signals to fine-tune growth and development.

## Materials and Methods

### Plant Materials

All plant material used were of the Col-0 ecotype. All mutants are combinations of the following alleles; *nf-yc3-1* (SALK_034838), *nf-yc4-1* (SALK_032163), *nf-yc9-1* (SALK_058903), *nf-yb2-1* (SALK_025666), *nf-yb3-2* (SALK_150879). The *p35S:NF-YB2* and *p35S:NF-YB3* lines and the various SALK insertion lines were all previously described [7], [12], [14], [16]. Introduction of native promoter genomic constructs into the *nf-yc triple* mutant demonstrate that *NF-YC3* and *NF-YC9* can rescue the early germinating phenotype on ABA media. As expected, *NF-YC4* does not rescue this phenotype (Figure S4), although it does rescue an *nf-yc triple* late flowering phenotype [16].

All germination and root growth assays were performed using seeds that were collected from plants grown concurrently in the same growth chamber under standard long day conditions (16 h day/8 h night, 90 µmol m^−2^ s^−1^, 22°C). Plants for matched seeds were grown in media containing equal parts Farfard C2 Mix and Metromix 200 supplemented with 40 g Marathon pesticide and dilute Peter’s fertilizer (NPK 20∶20:20).

### Germination and Root Growth Assays

Matched seed sets were harvested and allowed to after ripen for between 2 to 4 months before use in assays. Seeds were surface sterilized and plated onto Gamborg’s B5 media (SIGMA, St. Louis, MO, Cat#G5893) or B5 media containing the appropriate amount of (+/−) ABA (SIGMA, Cat#A4906). For germination and cotyledon greening assays, plates were cold stratified for 3 days and placed in continuous light at 90 µmol m^−2^ s^−1^ at 22°C in a Conviron model ATC13 chamber. Seeds were scored every 12 hours post incubation for visible radical emergence as a proxy for seed germination. Greening was assayed by counting plants with open green cotyledons on day 10 [51]. Plants for root growth assays were sown to B5 plates and incubated under long day conditions for four days before transfer to plates supplemented with ABA. Plates were oriented vertically for an additional seven days. All plates were photographed and primary root length was measured with image J [67]. All germination, greening, and root growth assays were repeated a minimum of three times with at least two independent sets of matched seeds with consistently similar results (see Figures S5–S7 for additional replicates). All statistics were performed in either INSTAT or Prism (GraphPad Software - La Jolla, CA).

### Promoter Analysis

Over representation analyses on nuclear encoded genes from the *nf-yc triple* were performed on genes misregulated ≤−1.5 and ≥1.5 fold (p<.05) compared to Col-0. Details of the microarray experiment and public access to MIAME compliant data were previously reported [16]. Because the purpose of the current analysis was hypothesis generation (which were later tested), we relaxed the stringency of the microarray analysis here by removing the Benjamini-Hochberg false positive correction. For MEME de-novo motif discovery, −500 bp of upstream sequence of the misregulated genes was obtained using the bulk data retrieval tool at The Arabidopsis Information Resource (TAIR, www.arabidopsis.org). Upstream DNA sequences were fed to the MEME program and analyzed using the following parameters: motif width between 5 and 12 bp, any number of repetitions of motifs, and search for up to 6 motifs. All other options were left as default (http://meme.nbcr.net/meme4_5_0/intro.html, [42]). Motifs discovered through MEME analysis were then compared against known transcription factor binding sites from Jasper, Transfac and Uniprobe using the TOMTOM motif comparison tool [43]. Positions of *CACGTG* motifs relative to the transcriptional start were adapted from the MEME analysis. The same misregulated gene list was input into the Athena analysis suite using a 500 max bp upstream cutoff and, otherwise, default settings (http://www.bioinformatics2.wsu.edu/cgi-bin/Athena/cgi/home.pl, [32]).

### Protein-Protein Interaction Network

Individual protein-protein interaction networks were built for NF-YC3, NF-YC4 and NF-YC9 using GeneMANIA (http://www.genemania.org, [44], [45]). Selection criteria to develop the network map in GENEMANIA were predicted interactions and physical interactions with a 50-gene output. Default settings in GeneMANIA were used for network weighting. The individual network maps built in GeneMANIA for NF-YC3, NF-YC4 and NF-YC9 were downloaded as text files and combined to build a protein interactome in Cytoscape 2.8.0 (http://www.cytoscape.org, [46]). Data from Y2H library screens and directed Y2H assays done in the Holt lab and published interactions of NF-YC3, NF-YC4 and NF-YC9 [16] were manually added to the protein interactome in Cytoscape. See File S1 for a fully annotated list of genes (with AGI numbers and references) used to build the protein interactome.

### RNA Isolation and qPCR

Total RNA was isolated from 20 mg of matched, stratified seeds using the E.Z.N.A. Plant RNA Kit per the manufacturer’s instructions for difficult samples (Omega Biotek, Inc., Norcross, GA, CAT#R6827-01). Prior to RNA extraction, seeds were sown on beds of Whatman paper saturated with liquid Gamborg’s B-5 media, cold stratified for 2 days, and exposed to 24 hours of continuous light at 22°C before harvesting. To completely remove genomic DNA, samples were DNAse treated on E.Z.N.A. RNA isolation columns (Omega Biotek, CAT# E1091). Quality and quantity of RNA samples were confirmed by spectrophotometry (Thermo Scientific, Waltham, Massachusetts, NanoDrop™ 1000). First-strand cDNA synthesis was performed using the Superscript III First-Strand Synthesis System (Invitrogen, Carlsbad, California, Cat#18080-051) with supplied oligo dT primers. qPCR was performed as previously described [14], except we used an Applied Biosystems Prism 7500 analyzer (Life Technologies, Carlsbad, California) and the Fermentas Maxima SYBR Green qPCR Master Mix (Fermentas, Glen Burnie, Maryland, Cat#K0222). For each genotype, we analyzed three independent, biological replicates in two separate experiments with similar results. All samples were normalized to the constitutively expressed gene At2g32170 as previously described [68]. Sample comparisons were performed by the 2^(*−*ΔΔ*C*^ *_T_* ^)^ method [69], and errors (standard deviation) were computed as previously described [70]. qPCR primers for *ABI3*, *ABI5*, *AIA*, *AIL*, *RAB18*, and *RD29B* (AT3G24650, AT2G36270, AT1G64110, AT3G17520, AT5G66400 and AT5G52300, respectively) were designed using Primer3 in Genious Pro 5.1.4 (www.genious.com, [71]). Primer sequences for qPCR and cloning are in File S2.

### DNA Manipulations

All target DNA fragments were generated by PCR using Pfu Ultra II (Agilent Technologies, Santa Clara CA, Cat#600670-51) and cloned into the Gateway™ entry vector pENTR/D-TOPO (Invitrogen, Carlsbad, California, Cat#45-0218). The full length coding regions of *NF-YB2*, *NF-YB3*, NF-YB10 *NF-YC3*, *NF-YC4*, *NF-YC9*, *ABF1*, *ABF2*, *ABF3*, *ABF4*, and *HY5* (AT5G47640, AT4G14540, AT3G53340, AT1G54830, AT5G63470, AT1G08970, AT1G49720, AT1G45249, AT4G34000, AT3G19290, and AT5G11260, respectively) were generated from Col-0 cDNA populations by standard methods. Partial clones of NF-YB2 and NF-YB10, as well as chimeric constructs between NF-YB2 and NF-YB10, were generated by PCR using Pfu Ultra II and cloned into the Gateway™ entry vector pENTR/D-TOPO. In partial clones that do not contain a native start codon an ATG was added in front of the region of interest. All constructs were sequenced and found to be identical to the expected sequences found at The Arabidopsis Information Resource database [72].

### Yeast Two-Hybrid Analyses

Gateway™ entry clones containing the full length coding regions of *NF-YC3*, *NF-YC4*, *NF-YC9*, *ABF1*, *ABF2*, *ABF3*, *ABF4*, and *HY5* were recombined using the LR Clonase II reaction kit (Invitrogen, cat#56485) into ProQuest™ Two-Hybrid System vectors pDEST22 and pDEST32 (Invitrogen, Cat#PQ10001-01). All interactions were tested per the manufacturer’s instructions. X-Gal assays were performed on nitrocellulose membranes containing yeast colonies frozen in liquid nitrogen and incubated at 37°C in Z-buffer containing X-Gal (5-Bromo-4-chloro-3-indoxyl-beta-D-galactopyranoside, Gold Biotechnology, St. Louis, MO, cat#X4281L). Y2H library screening with NF-YB2 and NF-YC9 was previously described [16] using published libraries [73]. These libraries were derived from mRNA extracted from flowers, siliques, seeds, and seedlings (including hormone treated).

### Gus Staining and Microscopy

All *pNF-Y::GUS* fusions used in seed coat expression assays were previously described [9]. Seed coats were dissected and GUS staining was performed as previously described [74], [75]. Seeds coats and embryos were visualized using a Zeiss AxioImager Z1 m with Apotome (Zeiss - Oberkochen, Germany), using the DIC/BF filter and recorded using onboard Axiocam MRm and MRm5 camera. Images are compressions of a 3D Z-stack into a 2D image using the deconvolution and extended focus feature in the Axiovision software (version 4.8.1).

## Supporting Information

Figure S1 **Protein alignment of NF-YB2, NF-YB3 and NF-YB10.** Full-length amino acid sequences for NF-YB2, NF-YB3 and NF-YB10 were aligned and visualized using ClustalW within the software package Geneious Pro5.6 (www.geneious.com). The junction site used to create chimeric constructs between NF-YB2 and NF-YB10 is annotated.
(TIF)

Figure S2 ***NF-YB*** **and** ***NF-YC*** **mutants show no significant differences in root growth on ABA.** Mutant lines were germinated and grown on B5 media for four days and then transferred to B5 media, B5 media +10 µM ABA, or B5 media +50 µM ABA and grown vertically for 7 days. Bars represent the mean primary root length (n ≥12 plants from 2 separate experiments). Error bars are 95% confidence intervals. No statistical significance between any samples on the same growth media was measured using ANOVA (p>0.05).
(TIF)

Figure S3 **Public microarray data visualized by eFP browser show** ***NF-YC3*** **and** ***NF-YC9*** **, but not** ***NF-YB2*** **and** ***NF-YB3*** **are expressed in seeds and during early germination.** Absolute levels for *NF-YC3*, *NF-YC4*, *NF-YC9*, *NF-YB2*, and *NF-YB3* were queried in the eFP browser with a signal threshold of 250 [78], [79]. Note that the lack of *NF-YC4* signal on public microarrays is likely due to problems with the probe (which is predicted to detect more than one gene) and not our GUS fusion. For example, according to public microarrays, *NF-YC4* is not expressed in leaf tissues, although we have previously published mRNA and protein data (using a native antibody) showing this is incorrect and there is a clear genetic requirement for *NF-YC4* in the leaf-initiated process of photoperiod-dependent flowering [16].
(TIF)

Figure S4 **Rescue of the** ***nf-yc triple*** **mutant early germination phenotype on ABA media.** Seeds were plated on media supplemented with 0.5 µM ABA as previously described (approximately 30 seeds/replicate, three replicates per line). Percent germination is shown at 84 hrs. These rescue lines were previously described and used to show rescue of the late flowering phenotypes of *nf-yc triple* mutants [16]. Asterisks represent significant differences in Fisher’s Exact Test comparing *nf-yc triple* mutants to all other lines (p<0.05).
(TIF)

Figure S5 ***NF-YC*** **mutants show opposing phenotypes in response to ABA.** Seeds were plated on media supplemented with 1 µM ABA as previously described and germination was scored every 12 hours. Each replicate of at least 30 seeds was independently graphed along with wild type controls for A) *nf-yc3,* B) *nf-yc4,* C) *nf-yc9,* D) *nf-yc3/c4,* E) *nf-yc3/c9,* F) *nf-yc4/c9,* and G) *nf-yc triple*.
(TIF)

Figure S6 ***NF-YC*** **mutants show opposing phenotypes in response to ABA.** Seeds were plated on media supplemented with 2 µM ABA as previously described and germination scored every 12 hours. Each replicate of at least 30 seeds was independently graphed along with wild type controls for A) *nf-yc3,* B) *nf-yc4,* C) *nf-yc9,* D) *nf-yc3/c4,* E) *nf-yc3/c9,* F) *nf-yc4/c9,* and G) *nf-yc triple*.
(TIF)

Figure S7 ***NF-YC*** **mutants show altered greening response to ABA.** Percentage of plants with open green cotyledons at 10 days for all combinations of *nf-yc* mutants. Each bar represents an independent replicate containing at least 90 plants. Three replicates for each genotype are presented.
(TIF)

File S1 **List of genes that interact with NF-YC3, NF-YC4 and NF-YC9.** AGI numbers are included and common names are given. Additionally the type of interaction (published physical interaction/predicted interaction) and the reference/source of information are given. The.xlsx file can be directly uploaded to Cytoscape (http://www.cytoscape.org/) to build a interactive protein interactome.
(XLS)

File S2 **List of primers used to generate all clones and perform qPCR experiments (Microsoft Excel format).**
(XLSX)
