# Supplementary figures and images for: NUCLEAR FACTOR Y Transcription Factors Have Both Opposing and Additive Roles in ABA-Mediated Seed Germination
Source: PLoS One. 2013 Mar 19;8(3):e59481. doi: 10.1371/journal.pone.0059481 (PMC3602376; doi:10.1371/journal.pone.0059481)

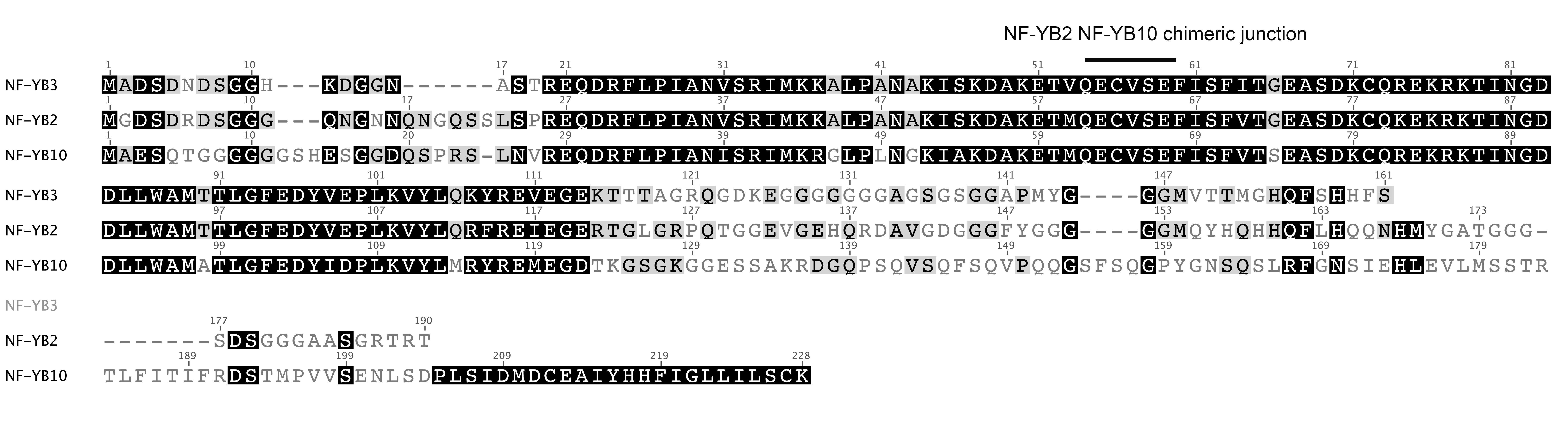

Supplement: Figure S1 — Protein alignment of NF-YB2, NF-YB3 and NF-YB10. Full-length amino acid sequences for NF-YB2, NF-YB3 and NF-YB10 were aligned and visualized using ClustalW within the software package Geneious Pro5.6 (www.geneious.com). The junction site used to create chimeric constructs between NF-YB2 and NF-YB10 is annotated. (TIF) [file pone.0059481.s001.tif]

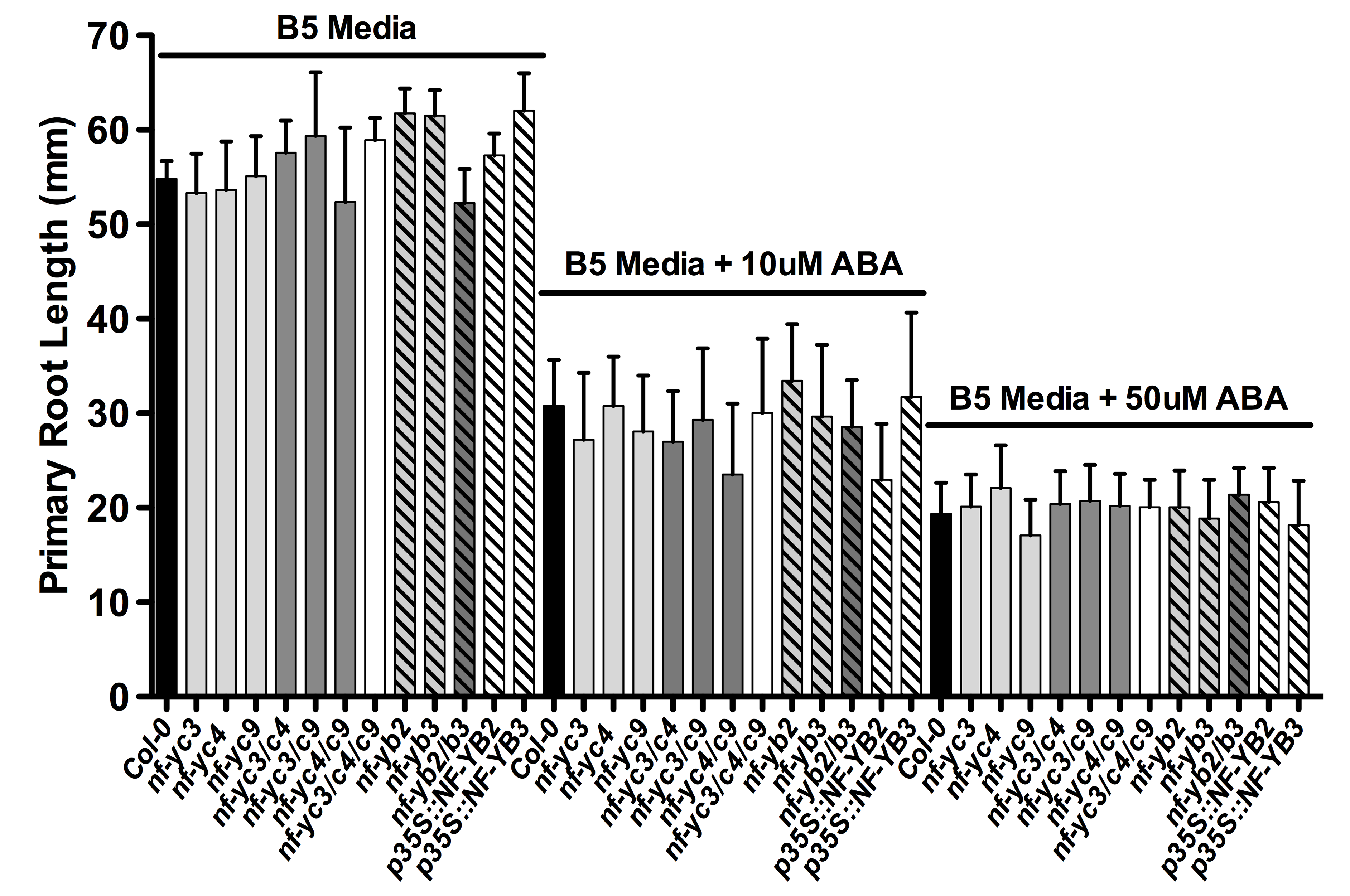

Supplement: Figure S2 — NF-YB and NF-YC mutants show no significant differences in root growth on ABA. Mutant lines were germinated and grown on B5 media for four days and then transferred to B5 media, B5 media +10 µM ABA, or B5 media +50 µM ABA and grown vertically for 7 days. Bars represent the mean primary root length (n ≥12 plants from 2 separate experiments). Error bars are 95% confidence intervals. No statistical significance between any samples on the same growth media was measured using ANOVA (p>0.05). (TIF) [file pone.0059481.s002.tif]

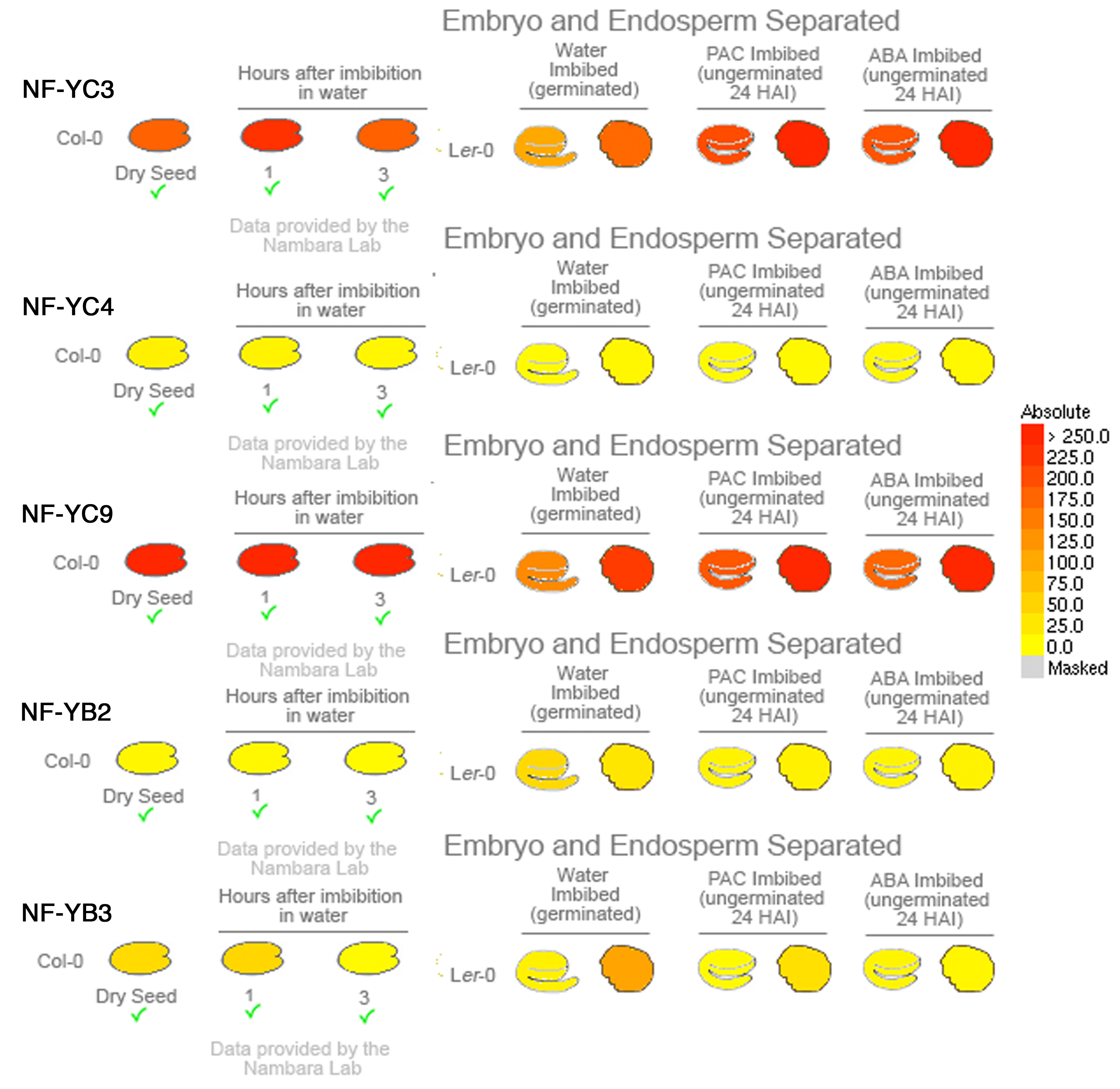

Supplement: Figure S3 — Public microarray data visualized by eFP browser show NF-YC3 and NF-YC9 , but not NF-YB2 and NF-YB3 are expressed in seeds and during early germination. Absolute levels for NF-YC3, NF-YC4, NF-YC9, NF-YB2, and NF-YB3 were queried in the eFP browser with a signal threshold of 250 [78], [79]. Note that the lack of NF-YC4 signal on public microarrays is likely due to problems with the probe (which is predicted to detect more than one gene) and not our GUS fusion. For example, according to public microarrays, NF-YC4 is not expressed in leaf tissues, although we have previously published mRNA and protein data (using a native antibody) showing this is incorrect and there is a clear genetic requirement for NF-YC4 in the leaf-initiated process of photoperiod-dependent flowering [16]. (TIF) [file pone.0059481.s003.tif]

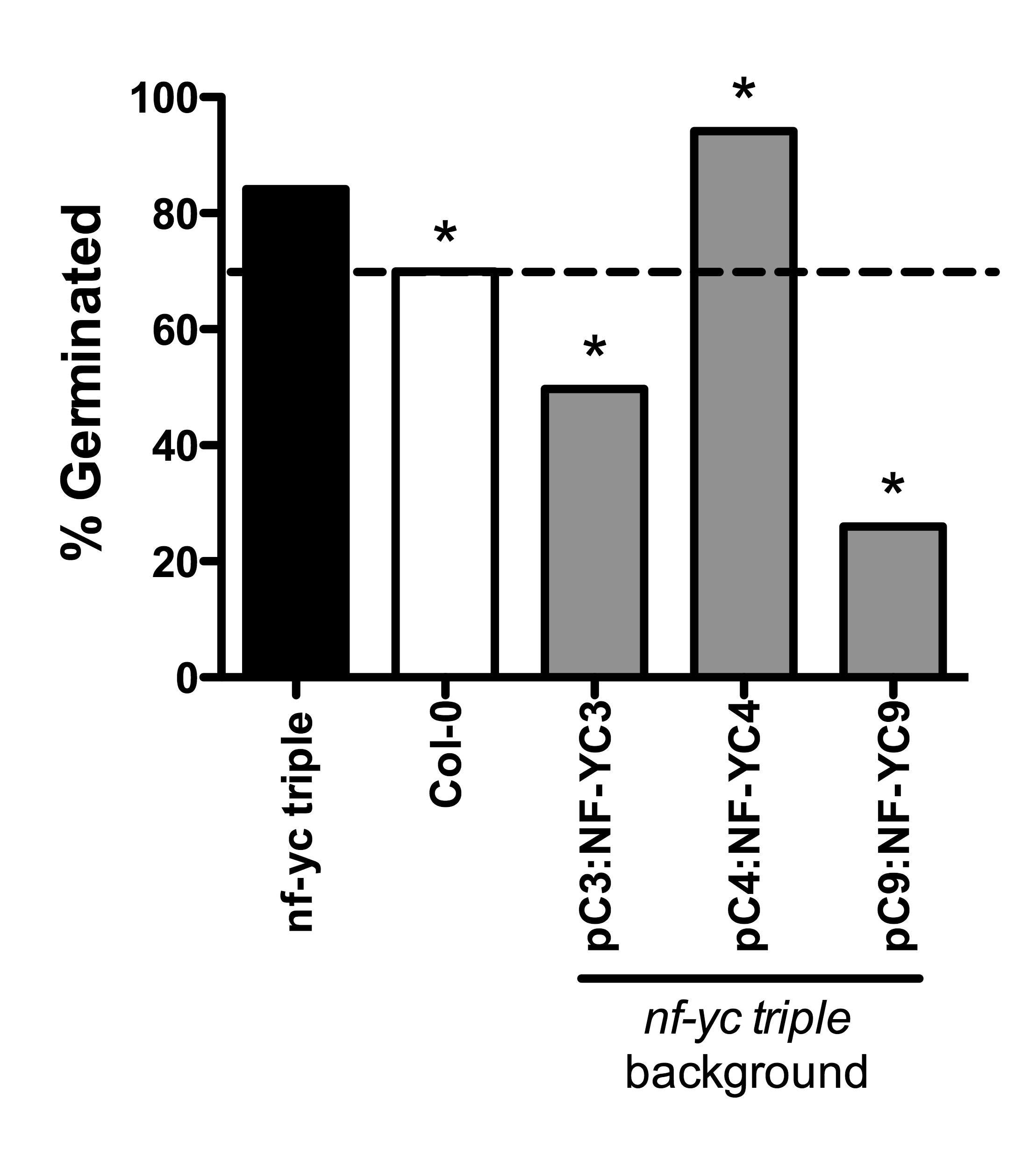

Supplement: Figure S4 — Rescue of the nf-yc triple mutant early germination phenotype on ABA media. Seeds were plated on media supplemented with 0.5 µM ABA as previously described (approximately 30 seeds/replicate, three replicates per line). Percent germination is shown at 84 hrs. These rescue lines were previously described and used to show rescue of the late flowering phenotypes of nf-yc triple mutants [16]. Asterisks represent significant differences in Fisher’s Exact Test comparing nf-yc triple mutants to all other lines (p<0.05). (TIF) [file pone.0059481.s004.tif]

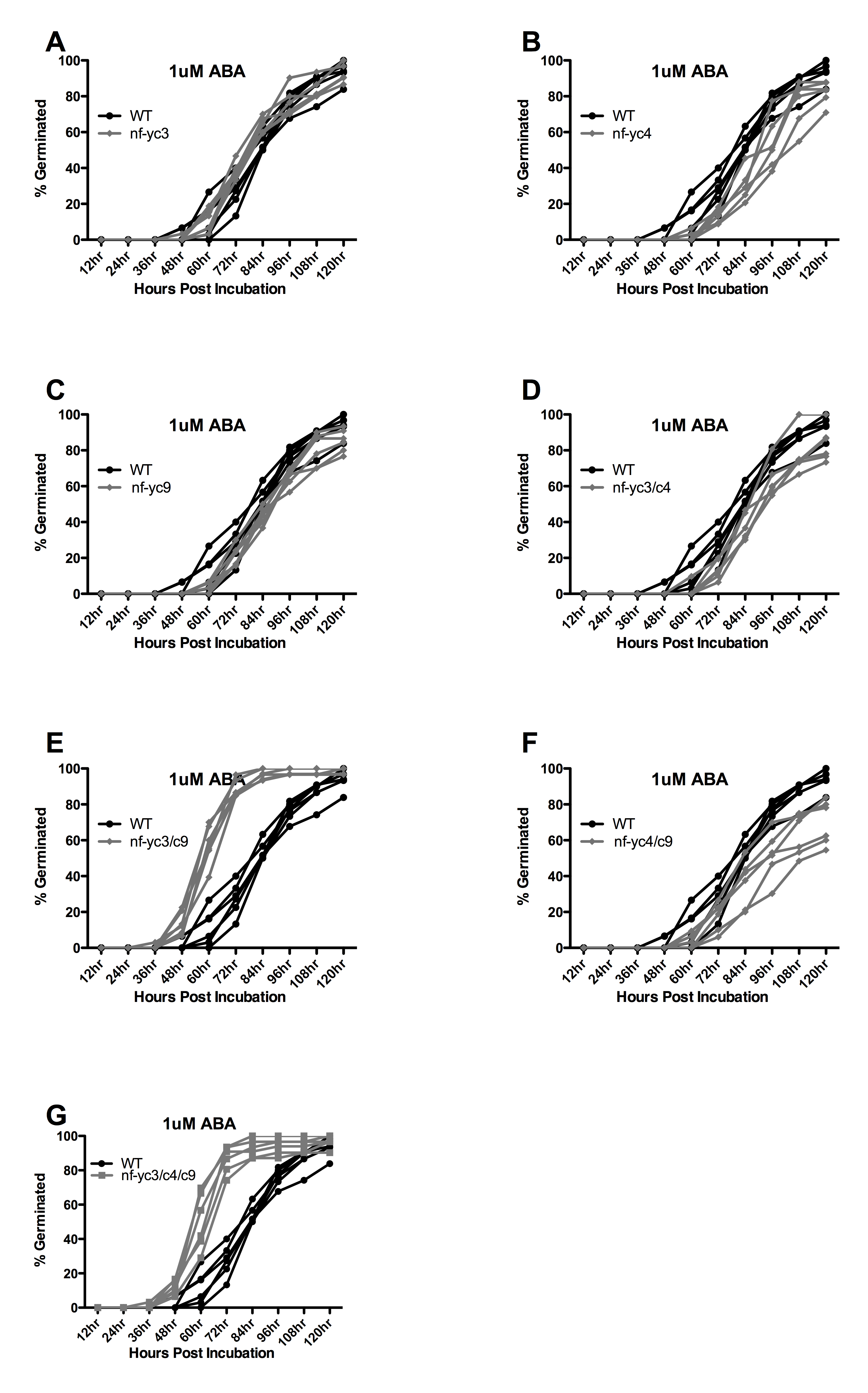

Supplement: Figure S5 — NF-YC mutants show opposing phenotypes in response to ABA. Seeds were plated on media supplemented with 1 µM ABA as previously described and germination was scored every 12 hours. Each replicate of at least 30 seeds was independently graphed along with wild type controls for A) nf-yc3, B) nf-yc4, C) nf-yc9, D) nf-yc3/c4, E) nf-yc3/c9, F) nf-yc4/c9, and G) nf-yc triple. (TIF) [file pone.0059481.s005.tif]

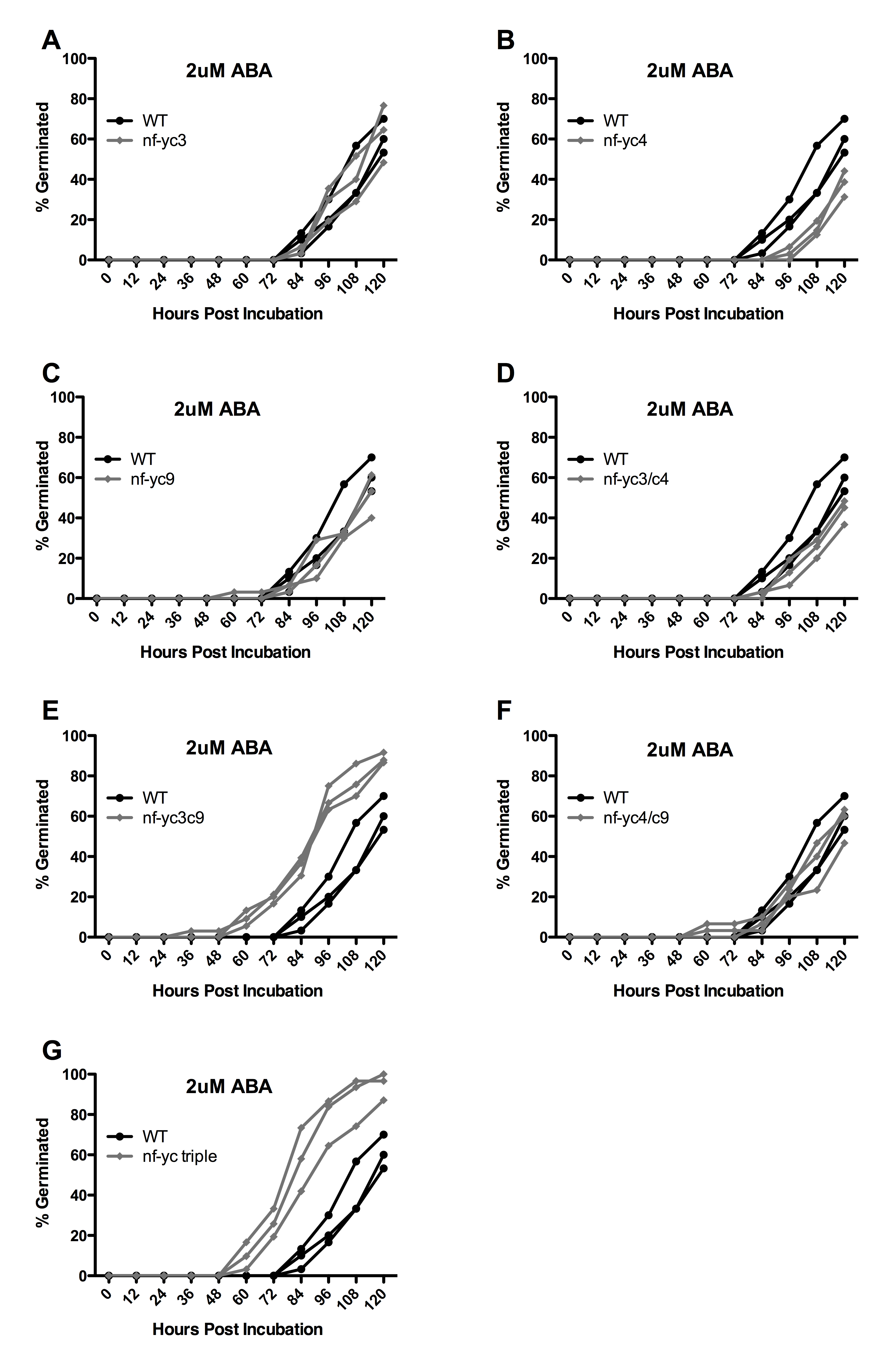

Supplement: Figure S6 — NF-YC mutants show opposing phenotypes in response to ABA. Seeds were plated on media supplemented with 2 µM ABA as previously described and germination scored every 12 hours. Each replicate of at least 30 seeds was independently graphed along with wild type controls for A) nf-yc3, B) nf-yc4, C) nf-yc9, D) nf-yc3/c4, E) nf-yc3/c9, F) nf-yc4/c9, and G) nf-yc triple. (TIF) [file pone.0059481.s006.tif]

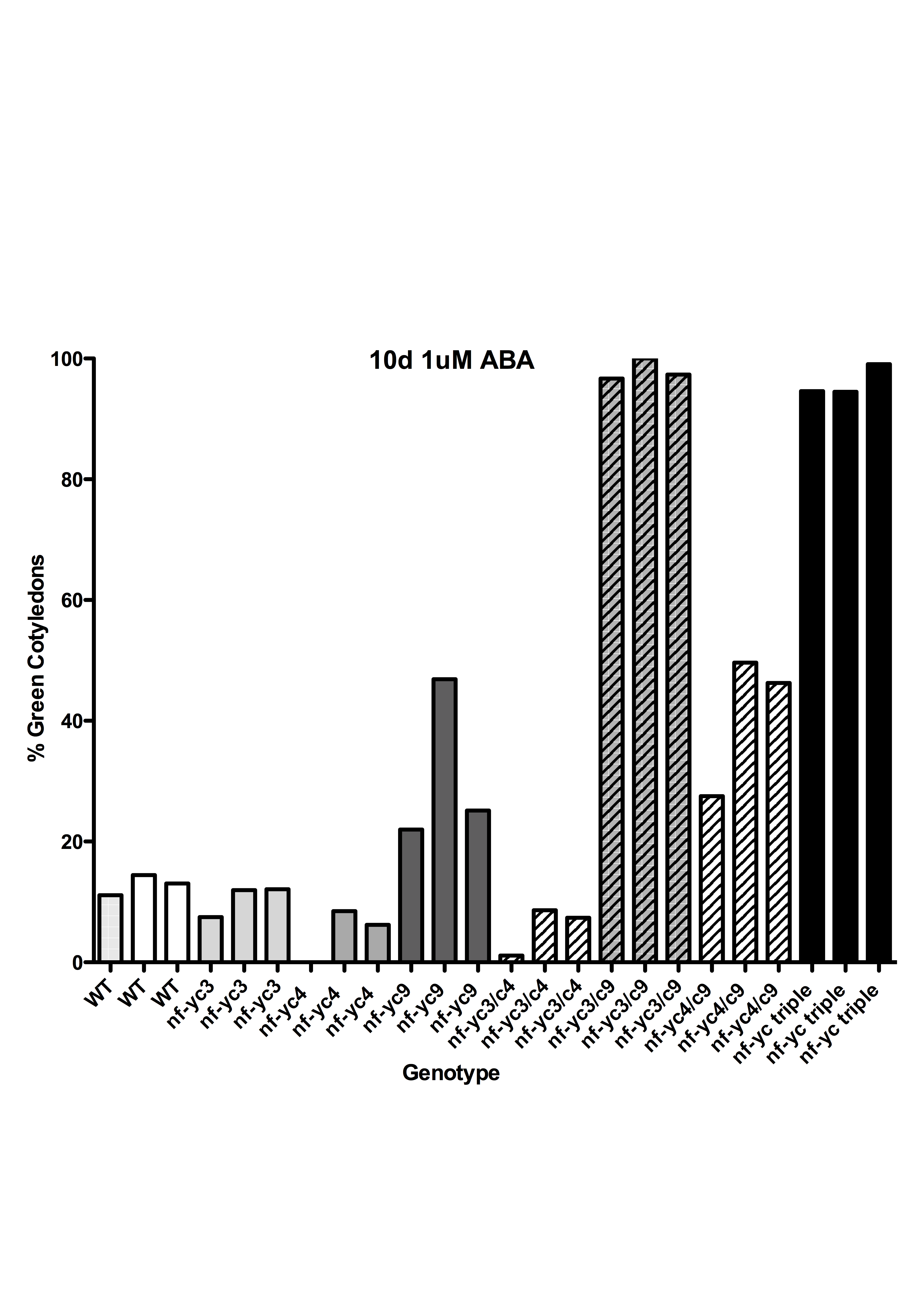

Supplement: Figure S7 — NF-YC mutants show altered greening response to ABA. Percentage of plants with open green cotyledons at 10 days for all combinations of nf-yc mutants. Each bar represents an independent replicate containing at least 90 plants. Three replicates for each genotype are presented. (TIF) [file pone.0059481.s007.tif]
